# Traditional health practitioners’ management of HIV/AIDS in rural South Africa in the era of widespread antiretroviral therapy

**DOI:** 10.1080/16549716.2017.1352210

**Published:** 2017-08-03

**Authors:** Thembelihle Zuma, Daniel Wight, Tamsen Rochat, Mosa Moshabela

**Affiliations:** ^a^ Africa Health Research Institute, Mtubatuba, South Africa; ^b^ School of Nursing and Public Health, University of KwaZulu-Natal, Durban, South Africa; ^c^ MRC/CSO Social and Public Health Sciences Unit, University of Glasgow, Glasgow, UK; ^d^ Human Sciences Research Council/Human and Social Development (HSD) and MRC Developmental Pathways to Health Research Unit, School of Clinical Medicine, University of the Witwatersrand, Johannesburg, South Africa; ^e^ Discipline of Rural Health, School of Nursing and Public Health, College of Health Sciences, University of KwaZulu-Natal, Durban, South Africa

**Keywords:** Traditional health practitioners, HIV/AIDS, ART, traditional and indigenous beliefs, traditional practices, KwaZulu-Natal, South Africa

## Abstract

**Background**: Traditional health practitioners (THPs) have been identified as a key local resource in the fight against human immunodeficiency virus/acquired immune deficiency syndrome (HIV/AIDS) in South Africa. However, their approaches to the treatment of people living with HIV (PLHIV) have been met with scepticism by some biomedical practitioners amid increasing access to antiretroviral therapy (ART).

**Objective**: In light of this ambivalence, this study aims to document and identify treatment approaches of THPs to the management of illness among PLHIV in the current era of widespread access to ART.

**Methods**: The study was conducted as part of a larger trial of Treatment as Prevention (TasP) in rural northern Kwa-Zulu Natal, intended to treat PLHIV regardless of CD4 count. Nine THPs were enrolled using purposive and snowballing techniques. Repeat group discussions, triangulated with community walks and photovoice techniques, were conducted. A thematic analysis approach was used to analyse the data.

**Results**: Eight of the nine THPs had received training in biomedical aspects of HIV. THPs showed a multilayered decision-making process in managing illness among PLHIV, influenced by the attributes and choices of the THPs. THPs assessed and managed illness among PLHIV based on THP training in HIV/AIDS, THP type, as well as knowledge and experience in the traditional healing practice. Management of illness depended on the patients’ report of their HIV status or willingness to test for HIV.

**Conclusions**: THPs’ approaches to illness in PLHIV appear to be shifting in light of increasing exposure to HIV/AIDS-related information. Importantly, disclosure of HIV status plays a major role in THPs’ management of illness among PLHIV, as well as linkage to HIV testing and care for their patients. Therefore, THPs can potentially enhance the success of ART for PLHIV when HIV status is known.

## Background

Sub-Saharan Africa continues to be a region heavily affected by human immunodeficiency virus/acquired immune deficiency syndrome (HIV/AIDS) [1–4]. In South Africa, significant developments have been made in managing the HIV/AIDS epidemic [5–7]. In 2004, South Africa saw the rollout of what would become the world’s largest antiretroviral therapy (ART) programme, resulting in significant improvements in morbidity and mortality, as well as improvements in quality of life [8,9]. The use of ART as prevention, by treating all patients infected with HIV irrespective of their CD4 count, has also contributed immensely to the most recent positive developments [10–14]. Growing evidence suggests that these innovations may significantly reduce HIV incidence at a population level [15–17]. As of 2016, South Africa is initiating ART with all patients testing HIV positive regardless of CD4 count, in line with the recommendations by the World Health Organization (WHO) [12]. However, other studies have suggested that, on their own, biomedical interventions are not enough to deal with the epidemic [18–21]. Structural and sociobehavioural factors have been considered major barriers to ART initiation [22,23].

While large numbers of people are being initiated on ART, several research studies have found that the use of traditional health practitioners (THPs) continues to delay individuals from timeously obtaining care, and remaining in care [24–27]. Furthermore, numerous studies have examined the implications of traditional medicines and practices related to HIV treatment and management [28–31]. The traditional healing system has been claimed to be illogical, lacking in both scientific validity and suitable policies for its products and practices [32,33]. However, other research suggests how THPs can be beneficial in the context of HIV/AIDS [34–37]. Indigenous belief systems drive significant levels of medical pluralism with the continued use of traditional healing alongside ART care, particularly in rural populations [38–40]. Even when people living with HIV (PLHIV) can access HIV treatment, they continue to use THPs to complement ART, and often find support from THPs and use traditional healing for reasons not directly related to the HIV [37,41,42]. Moreover, patients sought traditional care when they could not access ART [30,43].

While some studies have indicated that THPs lack the biomedical skills and knowledge to diagnose and treat HIV [34,35,44], THPs who are trained in HIV/AIDS have been used to educate patients about the causes and consequences of HIV, and have provided physical and psychosocial community-based HIV care [19,45–47]. A quantitative study conducted among patients consulting THPs in the context of HIV/AIDS found that PLHIV did so owing to several complex health situations [28]. These include THPs’ supportive role for encouraging safer sexual practices, HIV testing and treatment, and providing community-based HIV care [28]. In contrast, other studies have identified negative aspects of THP use in the context of HIV/AIDS, such as THPs lacking the skills and resources needed to manage HIV/AIDS [41,44,48–50]. Given the rapidly changing landscape of HIV care and management, it remains unclear how illness is managed by THPs among PLHIV.

The recent shift in HIV/AIDS care and treatment guidelines aims not only to use ART for the treatment of advanced HIV disease, but also to prevent disease progression in PLHIV [12]. Therefore, it is important to explore how THPs assess and manage illness in PLHIV, and how such practices complement or hinder the wider scale rollout of ART considering ongoing changes in HIV care and management. The objective of this study was to explore THPs’ perceptions and understandings of HIV/AIDS, and their approaches and practices in relation to illnesses of PLHIV, in rural South Africa [11].

## Methods

### Study design

The study used an exploratory qualitative approach, combining semi-structured repeat focus group discussions (FGDs) [51,52], community walks, photovoice techniques [53] and participant observation [54] to understand and document encounters between THPs and their patients during consultations relating to HIV/AIDS.

### Study setting

The study was conducted in Hlabisa subdistrict of rural northern Kwa-Zulu Natal, South Africa [11], as part of the ANRS 12249 Treatment as Prevention (TasP) trial, through the Africa Health Research Institute (AHRI). The trial investigated whether HIV testing of all adult members in a community, followed by immediate ART initiation for all HIV-infected participants regardless of immunological or clinical staging, will prevent onward transmission and reduce HIV incidence in this population, where an estimated 29% of adults aged 15–49 years and 0.5% of adults aged 50 years and older are infected with HIV [55,56]. Exposure to ART among all HIV-infected individuals in this community increased from 0% in 2004 to 31% in 2011 [56].

### Study sampling

Four THPs were identified and recruited through purposive sampling with the help of community liaisons, and a snowballing technique was used to recruit and enrol five further THPs. THPs were included if they resided within the TasP study area, were 16 years old and above, were willing to commit to the 18 month period prescheduled for data collection and provided informed consent to participate in audio-recorded repeat FGDs. Seven female and two male THPs were recruited, aged 24–60 years. All of the THPs who were approached to participate consented to do so during initial contact. Enrolled THPs knew each other before the study and resided within the same TasP trial cluster, covering a total population of 34,000 inhabitants [57].

### Data collection

TZ conducted four repeat FGDs with the same THPs in the local language of isiZulu. FGDs were conducted in a local church, and lasted for 60–120 min per session. A semi-structured topic guide (Appendix 1) was used by the investigator to facilitate FGDs. The data collected were triangulated with a community walk around the trial cluster and photovoice techniques. An overview of the methodological approach is outlined in Table 1.

**Table 1.** Methodological approach and traditional health practitioner (THP) attendance.

Meeting (M)/date	Activities/main topics discussed	Approach used	Attendance
M1 22/02/2013	M1 focused on types of healers available in the community, how THPs described their beliefs and how they carried out healing practices	Individual and group narratives	9/9
M2 30/05/2013	M2 focused on the healers’ perspective on HIV (what they thought were other people in the community as well as their patients’ thoughts about HIV), their own perspective on HIV testing and treatment, and what their role as healers was	Individual and group narratives	9/9
M3 31/07/2013	M3 focused on cultural and traditional healing practices that THPs perceived were barriers to and/or facilitators for HIV testing and treatment	Individual and group narratives	9/9
M4 09/10/2013	M4 was a community walk (CW). Guidelines for the CW included an introduction to the concept of using photographs to document parts of daily lives and perspectives. Healers were informed that the process would be undertaken as a group, and that they would lead the walk and use a digital camera. They were asked to identify and capture any image they considered a barrier to or facilitator for HIV testing and treatment, and treatment adherence in their community, to provide further insight into their lived experiences. A maximum of 20 photos per participant was allowed	Individual and group narratives, and community walk	8/9
M5 13/11/2013	M5 was a panel discussion about the images taken in M4. Each THP presented his or her own photos	Panel discussion	7/9

All discussions were transcribed verbatim, and translated from isiZulu to English by two trained translators who were native isiZulu speakers. Quality checks were conducted by the facilitator (TZ) to ensure completeness and accuracy of transcripts.

### Data analysis

Germond and Cochrane’s healthworld framework was used to explore how THPs understood and defined HIV/AIDS and what factors determined the practices and approaches they used to manage illness in PLHIV [58]. The healthworld framework recognizes that illness is a biological and a social phenomenon, needing both specialized ‘scientific’ healthcare and social and interpersonal meaning [59,60]. According to Germond and Cochrane, the scientific approach is characterized by a huge reliance on quantitative measures which recognize disease as a deviation from a biomedical norm. The social approach is characterized by an interest in the experience of illness [60]. The study used thematic analysis [61,62]. A coding framework was developed to code specific data regarding information on: (a) understanding of HIV/AIDS by THPs; (b) how THPs managed patients’ illness in the context of HIV; (c) and information related to HIV testing and ART. Data were coded, extracted and summarized in charts. Initial coding was followed by a joint repeated review of codes to develop thematic categories. These thematic categories were further refined and synthesized into emerging themes and subthemes, outlined in Table 2. Broad categories that emerged were related to THPs’ assessment of illness and THPs’ management of illness.

**Table 2.** Summary of findings: emergent themes and subthemes illustrated with quotations.

Category	Theme	Subthemes
Assessment	THP attributes	1. HIV/AIDS-related knowledge and information 2. HIV/AIDS training and exposure 3. Type of THP and experience
Management	THP choices	1. Use of ART and other medicines 2. Suitable medicines and rituals 3. Refusal to treat

THP, traditional health practitioner; HIV/AIDS, human immunodeficiency virus/acquired immune deficiency syndrome; ART, antiretroviral therapy.

## Results

### Characteristics of participants

THPs in the study described themselves as diviners*/Izangoma* (*n* = 6), herbalist*/Inyanga* (*n* = 1) and spiritualists*/Abathandazi* (*n* = 2), although they were not restricted to these roles and their healing practices were not mutually exclusive. A previous study with the same THPs described the processes followed in becoming a traditional healer and how these processes are related to THP roles [63]. THPs either were called by ancestors into their roles or underwent intensive training to learn about traditional medicines including plant, animal and mineral substances to provide healthcare. Most THPs used generic methods and practices to focus on the physical, spiritual, cultural, psychological, emotional and social elements of illness [63]. Eight of the THPs had been trained between 2005 and 2006 through the African Medical and Research Foundation (AMREF) on HIV/AIDS symptoms, counselling and home-based care for PLHIV [64]. AMREF trained about 82 THPs throughout the subdistrict [64]. The herbalist THP had not received any HIV-related biomedical training.

THPs identified different factors that shaped their diagnosis and management of patients.

### THP attributes

#### HIV/AIDS-related knowledge and understanding

Traditional forms of illness are understood to result from supernatural causes such as bewitchment or ancestral wrath [41,65,66]. Healing of illness largely involves divination, faith healing and traditional medicinal remedies [67–69]. When THPs explained their understanding of HIV/AIDS, they did not use explanations based on supernatural or spiritual dimensions. THPs understood HIV as a physical illness requiring biomedical treatment, not caused by the ancestors or bewitchment. THPs also understood that HIV was incurable and once people had acquired it, there was no way to reverse or cure the virus through rituals or traditional medicines. A predominant belief among all the THPs was that they could help patients with symptoms of HIV.

'P4: HIV is there and once someone has it there is no way they can go back and not have it anymore. Once it is there [in the body], it is there.' (Diviner, male, FGD 2)

It was acknowledged that HIV/AIDS could only be managed within the biomedical health system by using ART and adhering to treatment. THPs distinguished between illnesses treated within traditional and biomedical health systems, and accepted that HIV/AIDS could only be understood and treated within the biomedical system. THPs saw nothing problematic with patients’ engaging with biomedicine when it was necessary for the well-being of the patient, such as in the case of HIV/AIDS.

'P4: … the truth is that if you don’t take pills, you will eventually die so it is better that once you discover that you are positive, you need to accept the situation as it is otherwise you will die, but if you take pills, you stand a good chance of achieving more in life but if you decide to kill yourself because you are afraid of what people will say or think, people will never do anything for you and you just have to do things for your own sake.' (Diviner, male, FGD 3)

All THPs said that ancestors did not condone behaviour which made people vulnerable to HIV, such as having multiple sexual partners. Furthermore, ancestors did not protect people from acquiring HIV and once they had HIV, they needed to go to the clinic for care and treatment. THPs shared that their healing power was not self-determined but was derived from the strength of their relationship with ancestors. Diagnosis and treatment of patients was acquired through communication with the ancestors. HIV/AIDS required a biological process to be diagnosed and treated, and this was not in line with the treatment of psychosocial conditions involving spiritual aspects of illness in need of healing, which THPs could identify and treat.

'P1: No, they [ancestors] don’t protect you from such things as HIV.' (Faith healer, female, FGD 2)

#### HIV/AIDS training

THPs used HIV-related information acquired from AMREF HIV training to assess illness in their patients. The information was mostly related to identifying symptoms of HIV in patients, such as unexplained weight loss, coughing for long periods, sexually transmitted infections (STIs) and a combination of illnesses for a long period. While THPs were unable to diagnose HIV themselves, it was important for them to have HIV-related information, since this let them know how to manage and support patients whose illness symptoms were unclear or related to HIV.

The THP who had not received formal HIV training was considered HIV incompetent by fellow THPs in the study. They believed that untrained THPs were inefficient, could not offer referrals to biomedical facilities, and risked non-disclosure of HIV status, poor support to patients and poor management of HIV-related illness, for instance using inappropriate traditional healing methods. An example of this was expressed in the third FGD.

'P1: We as traditional healers who have been trained. We have learned that when a person comes to you at home what are you supposed to do and how are you supposed to treat that person. We did counselling so we know that we first need to sit down with a patient. I just wanted to explain to you what the difference is between trained and untrained healers.' (Faith healer, female, FGD 3)

THPs who had received formal training obtained certificates of competency from accredited training institutions [64]. These certificates did not mean that they were qualified biomedical practitioners but showed that they attended training to help them to identify certain diseases such as HIV/AIDS.

THPs also received HIV-related information from local clinics and research studies including the TasP trial. All the THPs in the study said that they had access to HIV-related information from a local primary health clinic. In addition, all the THPs resided in a community where AHRI had conducted two large HIV prevention trials between 2008 and 2014 [57,70]. During the trial, officers from the Community Engagement Unit of the AHRI and TasP trial nurses facilitated roadshows and community dialogues in all TasP trial clusters. Information provided during these community engagement events included how early HIV treatment could potentially reduce the risk of HIV transmission to an uninfected individual and improve long-term health outcomes for PLHIV; how the TasP trial would be conducted in the community, including details of the trial plan; and education on tuberculosis, and prevention of mother-to-child transmission of HIV (PMTCT). HIV-related training and information helped to improve the quality of health services provided by THPs.

'P7: … it was so helpful for me to be able to identify that the sickness of my initiates’ daughter was HIV related, and so I referred her to the clinic for testing, and the girl was 17 years old at the time ….' (Diviner/herbalist, female, FGD 3)

Undergoing formal training was regarded more credible than accessing or receiving information from the local clinic or research studies. THPs showed a special interest in participating in biomedical approaches to strengthen HIV/AIDS prevention, treatment and care. In general, THPs felt that recognizing the biomedical symptoms of HIV reduced the possibility of misdiagnosis during consultation with patients.

'P6: … my son in law just came to me and told me that he was suffering there was something wrong with his penis. He said he is coming to me because this thing will kill him and my daughter, then the family will not be able to look at each other. I said to him my child go to the clinic and check, he was tested positive. I told my daughter to go and check as well; they found out that there was nothing wrong, she was negative. I told her that she must always guard herself [use protection].' (Diviner/herbalist, female, FGD 4)

#### Type of THP

THPs also approached illness on the basis of their type of practice. Practices and methods varied between diviners, faith healers and herbalists. Diviners specialized in interaction with ancestors, faith healers in interaction with messengers sent from God, and herbalists in herbal remedies, although there was considerable overlap in their practices [63]. THPs reported that they combined such methods with HIV/AIDS-related information, training and experience to assess and manage illness. Images captured inside the THPs’ consultation rooms were used by THPs to narrate how patients were examined and how they sought further insight from ancestors to understand patients’ illnesses when the illnesses were not identified as being related to HIV/AIDS. This was expressed as stated below.

'P5: … when a person comes to me for the first time. They greet at the gate and I will stand up from where I am sitting in the house and go to them. I will check how sick that person is, if the person is very sick I will take my gloves and put them on, especially if that person has sores in the body, I put my gloves on before I help them. I will carry the person to the house. When we get there, I ask the ancestors to show me what is wrong with this person. When the ancestors come, I can also see with my eyes that there is something wrong. For example a person may tell me that he is sick, but when I look at my things I can see that yes he is sick but it is not the illness he is complaining about, he has a different illness.' (Diviner/herbalist, female, FGD 4)

The narrative above, demonstrated through an image taken during a community walk, indicates an approach used by a diviner/herbalist THP to assess the seriousness of illness in a patient during consultation. This demonstrates the combination of biomedical (using gloves/physical examination of the body) and traditional (communication with ancestors) approaches to healthcare used by THPs in the context of HIV/AIDS, regardless of THP type.

### THP choices

#### Use of ART and other medicines

THPs were not opposed to use of ART, and they negotiated traditional healing with patients who were already on ART, advising them that they would only prescribe traditional medicines that were not strong or at a lower dosage to avoid potential drug interactions. In some cases, THPs found that they were consulted by individuals who had just started ART and were not seeing any improvement in their health. THPs explained to patients that they could use both traditional and western medicines even while on ART. However, THPs stated that it was important that they first determined the effectiveness of ART among their patients before prescribing traditional medicines, particularly since the patients had just started using ART and issues of possible drug interactions were unknown. In these cases, THPs suggested to their patients that using ART alone could be sufficient to alleviate their illness, but THPs needed sufficient time to observe patient improvements. As a result, THPs achieved ongoing monitoring of patient outcomes. Images of different traditional medicines that could be used together with ART were presented by THPs in the fourth FGD.

'P9: … Even though he/she has been told about being positive, he is still going to live but he must stick to his treatment. He will continue using the treatment and also continue seeing me. I will tell him that I will not give him *imbiza* [concoction] because it is strong, we must first see how the treatment [ART] is working. At the end he will see that the treatment from the clinic is working and he will come back to thank me for encouraging him.' (Diviner, female, FGD 4)

On occasions where traditional medicines were prescribed in addition to ART, THPs encouraged patients not to discontinue using ART. Only medicines considered suitable to be used alongside ART were prescribed concurrently, based on the potency of such medicines. While THPs agreed that some people needed western and traditional medicines concurrently, they emphasized that they were not to be administered at the same time, and thus patients were cautioned to use treatments at different times of the day, and this view was shared by all THPs.

'P2: … [one] should give the doctor’s medicine or the clinics medicine its own space. When that has worked in the system, then [one] can administer the traditional medicine. It will also work in the system and [one] will go back to using the doctor’s medicine.' (Diviner, female, FGD 4)

#### Suitable medicines and rituals

THPs in this study recognized the complexity of treating HIV, and described the virus as elusive and not easy to target in the body. They recognized this as one of the reasons they were not able to treat or cure the virus. One of the healers stated:

'P9: The only problem with HIV is that it runs in the whole body. It runs through the blood and not just in one place that we can target …. That’s why traditional healers are failing to treat this disease.' (Diviner, female, FGD 3).

Nevertheless, THPs said that they prepared traditional medicines or prescribed rituals which were not harmful. For example, they explained that they used different medicines which they prescribed or administered to patients. THPs described these medicines as harmless to both those who were HIV positive and those of unknown HIV status.

'P9: … So I prepare something weak, a mixture of water and soil or salt. I look at how sick the person is. If he/she is very sick, I make *isiwasho* [sanctified water] and give it to them.' (Diviner, female, FGD 4)

THPs acknowledged that they were unable to target a virus inside the blood. However, they were able to offer rituals to cleanse the body symbolically. Sanctified water focused on symbolic cleansing and purification of the body to break any psychosocial and spiritual factors that might be connected to or a part of the patient’s illness.

THPs said that they withheld or limited treatment to people who presented with HIV symptoms, whose HIV status was unknown and if their condition could not be identified during divination, irrespective of the patient’s explanation.

'P2: … people come when they feel that they are sick. When they come, you can hear that when they cough they run out of breath. Usually I do tell them that they should also go to the clinic because I can see that what they have needs a clinic. I tell them to go to the clinic. A person will say no please give me the medicine; I will go to the clinic afterwards. But I refuse; I say no, please go to the clinic and get tested first.' (Herbalist, female, FGD 4)

#### Refusal to treat

It was also found that THPs decided not to treat some patients, and this was specifically the case when a patient showed HIV-related symptoms but did not disclose their HIV status to the THP. THPs said that they referred patients to the local clinics or hospitals if they could not help them.

'P6: … I only use ashes that they can lick and ask them to go to the clinic first. I tell them that once they have been to the clinic to test, they should come back with their results then I will be able to help them further, I will do the incisions. Others do go to the clinic, others do not go.' (Diviner/herbalist, female, FGD 4)

Disclosure of HIV status determined which traditional medicines could be prescribed or prepared, and which traditional rituals could be performed. For example, THPs did not prescribe potent traditional medicines for PLHIV. Potent traditional medicines, with potential for harm in weak patients, were those considered to cause diarrhoea and vomiting. Rituals such as skin incisions were also avoided among PLHIV.

Other factors related to THPs’ refusal to treat PLHIV included their lack of training in HIV/AIDS and lack of standardized procedures to handle HIV/AIDS. THPs reported that these factors often led to their feeling judged by the biomedical system for being responsible for the spread of HIV/AIDS; as a result, they would refuse to treat someone living with HIV.

'P7: … But right now since we are all traditional healers we can’t agree on one correct way of doing this. You see when you [biomedical practitioners] are training us you are saying that the HIV virus is spreading hugely like this because of us, the traditional healers. You are saying like that because whenever you train people you don’t invite even one of us to participate in that training ….' (Diviner/herbalist, female, FGD 3)

## Discussion

For THPs in this study, decisions around managing illness in PLHIV were complex and multilayered. THPs assessed and managed illness in PLHIV by both using HIV-related knowledge and communicating with ancestors. On the whole, THPs did not have standardized procedures to follow when managing illness in PLHIV, resulting in varying approaches dependent on the THP’s healing practice, HIV/AIDS training, THP choices and recommendations to patients. These findings build upon the results of a THP study conducted in the Eastern Cape Province of South Africa, which demonstrated that THPs did not follow specific procedures when treating PLHIV. Their methods generally involved divination, spiritual care, treatment of symptoms and referrals for biomedical care [71].

In this study, we found that HIV/AIDS was perceived by THPs as an incurable and complicated disease, which could not be treated by performing rituals or cured using traditional medicines. The current literature suggests a mixed account of THPs’ knowledge and understanding of HIV/AIDS. Some studies have found that THPs consider HIV to be caused by a combination of physical, environmental and spiritual impurity [72,73]. THPs who held this perspective also said that they could treat HIV-related symptoms such as swollen lymph nodes, mouth and genital ulcers, weight loss and diarrhoea [72]. A study conducted in KwaZulu-Natal found that, out of 11 THPs participating in the study, none said that they could cure HIV. A predominant belief among these THPs was that they could help patients with symptoms of HIV [41]. Other studies had previously suggested that THPs thought they could cure HIV/AIDS through different rituals and healing practices [49,65,74,75]. These findings could be reflecting contrasting views among THPs, but are most likely explained by a shift in the knowledge shared by some THPs concerning HIV/AIDS and treatment methods, particularly as the epidemic matures and knowledge continues to permeate society and local communities.

THPs in this study thought about the management of illness in PLHIV in varied ways, using both biomedical and sociocultural accounts to explain their understanding of HIV/AIDS and their responses to patients’ healthcare and treatment. We used the healthworld framework to explore how THPs think about HIV/AIDS, and define and respond to it, in relation to managing illness in PLHIV. We explored the interactions between biomedical ‘scientific’ and sociocultural approaches used by THPS. We highlight the biomedical and socially constructed healthworlds used by THPs to respond to and support PLHIV. Healthworlds are defined by Germond and Cochrane as relying on quantitative measures of biomedical definitions of disease; these are characterized by physical aspects of disease and they concentrate on pragmatic aspects of ill-health. In addition, healthworlds rely on socially constructed experiences and explanations of illness, concentrating on spiritual and psychosocial aspects of ill-health [58,76].

THPs focused on aspects of health covering religion, and physical, social and emotional well-being. We found that THPs approached HIV/AIDS based on HIV-related information and training. Moreover, THPs put a strong emphasis on the use of biomedical ‘scientifically’ appropriate methods for the diagnosis, treatment and care of HIV/AIDS. Research studies conducted with THPs have shown a marked increase in HIV/AIDS-related training and collaboration with the biomedical system [49,64,77]. Nevertheless, precise data are lacking on the total number of THPs who have been trained, and the effectiveness of that training, particularly given the changing epidemic of HIV [74,78–80] and the dynamic relationship between the traditional and biomedical health systems [81–83]. Evaluations of interventions to educate THPs in STIs and HIV have been limited by self-report measures and small samples [44,84,85]. As such, THPs’ understanding of HIV/AIDS remains contested. Previous studies with THPs have illustrated their potential interest in participating in biomedical approaches to strengthen HIV/AIDS care and prevention [64,86]. However, a study conducted in KwaZulu-Natal, South Africa, indicates that partnerships between the two health systems are far from being achieved [87].

THPs were cautious about treating patients who did not disclose their HIV status during assessment of their illness, and they said that such patients were referred to local clinics. Referral of PLHIV from THPs has been well documented in the literature [28,44]. Disclosure of HIV status determined which traditional medicines could be prescribed or prepared, and which traditional rituals could be performed. With South Africa adopting WHO recommendations to initiate patients on ART regardless of CD4 count [12,57], their quality of life has improved, they are experiencing fewer symptoms and HIV is no longer considered a terminal illness; rather, it is now listed as a chronic, manageable illness [8,88]. Thus, THPs miss PLHIV if they are asymptomatic or do not disclose their HIV status. These individuals may be prescribed medicines or rituals which are not appropriate for their condition. This finding sanctions the need for new ways of thinking about how THPs could be engaged, and how their roles could be incorporated and enhanced in order to offer quality care to patients subscribing to both healthcare systems in the era of wide ART availability.

In addition, THPs used approaches including divination, traditional medicines and rituals to manage aspects of patients’ illness that required psychosocial and spiritual healing. For example, THPs said that they offered cleansing rituals to symbolically cleanse patients so that they could break psychosocial and spiritual factors connected to their illness. The combination of biomedical and traditional healing approaches used by THPs, whether effective or not, demonstrates that PLHIV will seek care from both biomedical and traditional health systems, irrespective of HIV status and use of ART.

A recent scoping review notes how the social power existing among practitioners, in both the biomedical and traditional healing systems, may deprive patients of their views, values and choices of healthcare [82]. As the South African Department of Health continues to facilitate the formal registration and inclusion of THPs as key players in HIV/AIDS healthcare, there will need to be improved communication and understanding between the two health systems. It is suggested that creating common understandings and goals for collaboration is essential, and advancing the HIV response in sub-Saharan Africa will be likely to be restricted if the full engagement of THPs is not part of the developments [19]. Successful treatment of HIV/AIDS largely depends on both systems understanding contextual and interrelated factors, as this plays a role in ART adherence for individuals using both systems alternatively or concurrently [30].

### Limitations

This study is based on a small sample of only nine THPs, of whom six practised as diviners, two as spiritualists and one as a herbalist. All the THPs in the study resided in the same community, and therefore knew each other. This could potentially lead to social desirability bias leading THPs to say what was socially acceptable rather than what they individually did in practice, owing to the sensitive nature of the subject of HIV/AIDS and traditional healing. Most THPs (seven out of nine) were women. A larger and more inclusive sample may have enabled a comparison of THP approaches across different types and genders. There was an imbalance in the number of times the different participants’ quotations were used in the findings. This was due to some participants being more dominant and forthcoming, vocalizing their knowledge and expertise more than others. As such, their views and opinions dominated the discussions. The one THP who had not received HIV training did not respond to the others’ criticism that she was ‘HIV incompetent’, and the study did not elicit how this critique affected her professional identity or how her lack of training affected her management of patients with potentially HIV-related illness.

Since eight of the nine THPs had received HIV training from AMREF, the findings cannot be generalized to THPs who have not received HIV training. Moreover, THPs in this community were exposed to two large HIV prevention trials where they obtained additional HIV/AIDS information and knowledge. Findings have been interpreted in light of these factors. It is also important to note that the training took place between 2005 and 2006. Taking into account the developments in HIV/AIDS treatment and care, this training and knowledge held by THPs could be considered outdated and insufficient. More generally, our understanding of how these healers managed the HIV-related conditions of their patients would have been enhanced by having more information on their broader cosmologies and beliefs about illness causation, the commercial dimension to their practice and their relationships with their patients outside their professional roles.

## Conclusion

THPs in this study acknowledged that HIV/AIDS exists, but they did not treat HIV/AIDS and lacked standard practices or methods to handle the condition. THPs managed other illnesses in PLHIV and used their own judgement to weigh up the risks and benefits for patients. They primarily relied on symptoms to manage their patients; however, with the widescale rollout of ART, the symptoms that most patients presented with did not relate to HIV. Consequently, THPs were only partially equipped to manage illness in PLHIV in the ART era. Eight of the nine THPs in this study had received HIV-related training from AMREF in 2005. Even so, without accurate evaluation, the significance of such initiatives should be interpreted with caution. THPs emphasized the value of HIV/AIDS-related training in their practice, and investments in THP training on updated HIV/AIDS treatment and care should be considered. Understanding the role of THPs across the treatment cascade can essentially inform future interventions to improve patient outcomes, particularly in the era of TasP, as it raises clinical and pharmacological concerns.

## Paper context

We explored traditional health practitioners’ (THPs’) management of illness among people living with HIV in the era of widespread antiretroviral therapy, as it relates to developments made by the South African health service to advance HIV/AIDS management. We found that THPs are key players in HIV/AIDS care and there needs to be improved understanding between traditional and biomedical health systems. Further studies are needed to understand the role of THPs across the HIV/AIDS treatment cascade to inform future interventions to improve patient outcomes.

## Appendix 1. Topic guide for group discussions with THPs

**Topic guide for group discussions with THPs**

**Meeting one: Healthcare access in community**

- In this community, what types of traditional healers do you have?
- How does a person become a traditional healer?
- How do different traditional healers work?
- What kind of healthcare do people get from traditional healers?
- What is the role of healers in healthcare?

**Meeting two: Understanding of HIV and TasP**

- What are the benefits of treating people with HIV with ARVs?
- What would challenge HIV-infected people to start taking ARV treatment?
- Do traditional healers have an approach to HIV?
- If people thought that there were significant benefits in accessing early HIV treatment, do you think that they would go to testing and treatment centres? Why? Why not?
- What can we do to deal with the challenges of taking treatment?

**Meeting three: Local cultures to support regular and repeat testing**

- What elements of culture or traditions (as people in this community) do you think play a role in facilitating or hindering HIV testing, treatment and adherence?
- Do you think there is any alternative support to help deal with these culture-bound issues?

**Meeting four: Panel discussion of photos**

- Can each person discuss the photos that they took during the community walk, to share with the rest of the group, using photos to explain, what are the facilitators and/or barriers of HIV testing, treatment or adherence in this community?
